# Transcriptional profiling of single tumour cells from pleural effusions reveals heterogeneity of epithelial to mesenchymal transition and extra‐cellular matrix marker expression

**DOI:** 10.1002/ctm2.888

**Published:** 2022-07-10

**Authors:** Moen Sen, Ryan M. Hausler, Keely Dulmage, Taylor A. Black, William Murphy, Charles H. Pletcher Jr, Ling Wang, Chang Chen, Stephanie S. Yee, Scott J. Bornheimer, Kara N. Maxwell, Ben Z. Stanger, Jonni S. Moore, Jeffrey C. Thompson, Erica L. Carpenter

**Affiliations:** ^1^ Department of Medicine, Division of Hematology and Oncology, Perelman School of Medicine University of Pennsylvania Philadelphia Pennsylvania USA; ^2^ Life Science Innovation BD Technologies and Innovations Durham North Carolina USA; ^3^ Department of Pathology and Laboratory Medicine, Perelman School of Medicine University of Pennsylvania Philadelphia Pennsylvania USA; ^4^ BD Biosciences San Jose California USA; ^5^ Department of Medicine, Division of Gastroenterology, Perelman School of Medicine University of Pennsylvania Philadelphia Pennsylvania USA; ^6^ Department of Medicine, Division of Pulmonary, Allergy and Critical Care Medicine, Thoracic Oncology Group University of Pennsylvania Perelman School of Medicine Philadelphia Pennsylvania USA

Dear Editor,

Malignant pleural effusions (MPE) in advanced non‐small‐cell lung cancer (NSCLC) offer a rich source of tumour‐derived material for liquid biopsy.^1^ However, molecular monitoring of NSCLC is largely dependent on tumour biopsies. Previous NSCLC MPE studies either did not transcriptionally evaluate the tumour cell compartment of MPEs^2^ or relied on a positive selection of epithelial (EPCAM expressing) cells.^3^, ^4^ This strategy excludes cells transitioning to an invasive, mesenchymal phenotype through epithelial to mesenchymal transition (EMT).^5^, ^6^, ^7^ Here, we molecularly characterize single EPCAM‐negative and ‐positive MPE tumour cells (TCs) to investigate the potential of an MPE liquid biopsy.

Our study included 11 MPEs from nine NSCLC patients (Table 1 and Supporting Information). 1468 single TCs and 131 pools of 10–15 white blood cells (WBCs) were identified by flow cytometry [median of 146 TCs per patient (range 48–230)] (Figure 1A).^8^ Among 584 TCs passing quality control (QC), 483 completed staining for EPCAM, revealing that 67% (322 of 483) were EPCAM‐negative (Figure 1B). The proportion of EPCAM‐positive TCs ranged considerably from patient to patient (median 24%; range 0% ‐ 80%). Importantly, UPENN‐1 had no detected EPCAM‐positive TCs. This suggests that EPCAM based TC isolation may under‐represent the number and phenotypic diversity of TCs. t‐distributed stochastic neighbour embedding analysis revealed that TCs clustered away from WBCs (Figure 1C). Index sorting linked the transcriptional profile of each cell to its protein expression, demonstrating that cells in the WBC cluster were EPCAM‐negative but CD45‐positive (Figure 1C). We confirmed high expression of tumour specific genes *KRT7* and *KRT8* and epithelial gene *EPCAM* among cells in the TC but not the WBC cluster (Figure S1).

**TABLE 1:** Clinical characteristics for nine patients from whom 11 PE samples were obtained

**Patient**	**Histology**	**Sex**	**Race**	**Age at diagnosis (years)**	**Weeks on therapy at time of PE**	**Driver mutations**	**Therapy at time of PE**	**Smoking Status**
UPENN‐1	Adenocarcinoma	F	White	56	4	EGFR ex19 del	Chemo+ Avastin	Former
UPENN‐2	Adenocarcinoma	F	Asian	70	19	EGFR L858R	TKI + Avastin	Never
UPENN‐3A	Adenocarcinoma	F	White	64	1	BRAF V600E	Chemo	Former
UPENN‐3B	Adenocarcinoma	F	White	64	1	BRAF V600E	IO	Former
UPENN‐4	Adenocarcinoma	F	White	53	11	EGFR ex19 del	TKI + Avastin	Never
UPENN‐5A	Adenocarcinoma	F	White	78	9	None detected	IO	Current
UPENN‐5B	Adenocarcinoma	F	White	78	17	None detected	IO	Current
UPENN‐6	Adenocarcinoma	M	White	74	2	KRAS G12C	IO	Former
UPENN‐7	Adenocarcinoma	F	White	64	12	EGFR Exon 18 p.E709_T710delinsA	TKI	Never
UPENN‐8	Adenocarcinoma	F	White	55	36	EGFR ex19 del	TKI + Avastin + Chemo	Former
UPENN‐9	Adenocarcinoma	F	White	55	3	EGFR L858R	TKI	Former

Abbreviations: IO, immunotherapy; TKI, tyrosine kinase inhibitor.

**FIGURE 1:**
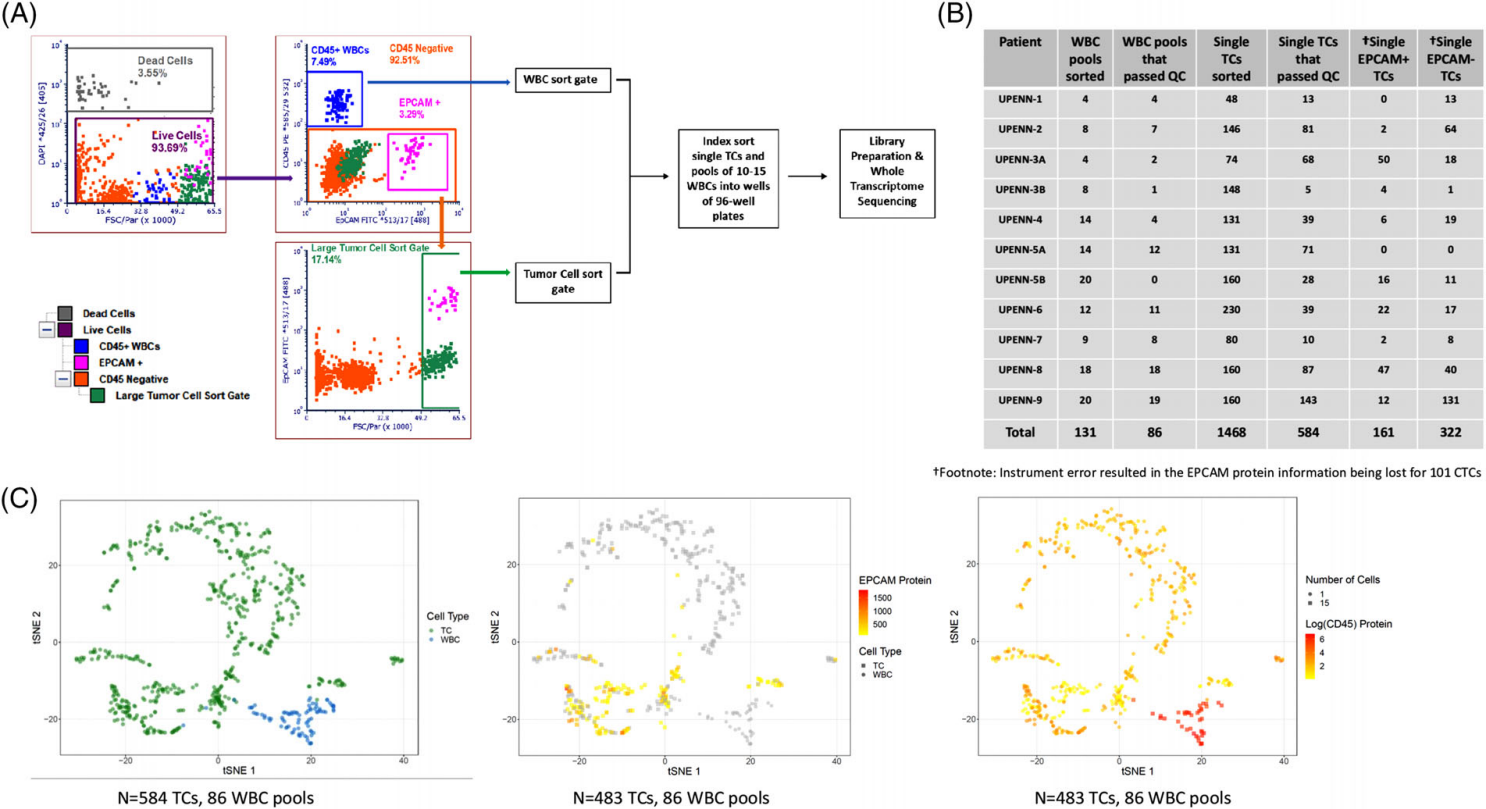
Isolation and characterization of pleural effusion tumour cells (TCs) and WBCs by single‐cell RNA sequencing**. (**A) Representative scatter plots demonstrating the flow cytometric gating strategy for the detection of TCs in the pleural effusion sample from patient UPENN‐9. 1468 single TCs and 131 pools of 10–15 WBCs from 11 malignant pleural effusions (MPE) samples were index sorted into 96 well plates for whole transcriptome RNA sequencing. B) Number of TCs and WBC pools that were sorted and passed QC are shown. C) t‐distributed stochastic neighbour embedding (t‐SNE) analysis of gene expression of 584 TCs with recorded EPCAM protein expression (483 TCs) and WBCs (86 pools) coloured by cell type (left), log10 mean fluorescence intensity (MFI) of EPCAM (TCs: square, WBCs: circle) (middle) and CD45 (right) shows WBCs cluster away from TCs and TCs have a heterogenous expression of EPCAM. Cells in grey are negative for EPCAM (middle) or CD45 (right) protein expression respectively. 18% (89/483) of TCs express CD45, albeit at 5.6 fold lower MFI than WBCs, consistent with previous studies demonstrating the occurrence of CTCs expressing leukocyte markers in patients with solid tumours^10^

We performed differential gene expression analysis to identify TC specific genes. 185 genes were significantly differentially expressed in MPE TCs versus WBCs (adjusted *p*‐value [*p*‐adj] <0.05 and log2 fold‐change log2FC>1.5; Figure S2A and Table S1). Genes significantly upregulated in TCs include NSCLC tumour markers *NAPSA*, *SFTPB*, *CEACAM6*, *C3, KRT7*, *KRT18*, and *KRT1* (Figure S2B). Gene Ontology (GO) revealed enrichment for gene signatures including extracellular matrix structural constituent (Figure S2C and Tables S2–4). Expression of tumour markers and lack of expression of immune markers suggest the lung tumour origin of the MPE TCs.

We sought to identify differentially expressed genes between EPCAM‐positive and EPCAM‐negative TCs. Sixty one genes were significantly differentially regulated in EPCAM‐positive TCs versus EPCAM‐negative TCs (*p*‐adj <0.05 and log2 fold‐change log2FC>1.5; Figure 2A and Table S5). Epithelial cell transcripts *MUC1*, *KRT7*, *CEACAM6* and *NAPSA* were significantly enriched in EPCAM‐positive TCs versus EPCAM‐negative TCs (Figure 2A) and expressed in the majority (62%–75%) of EPCAM‐positive TCs (Figure 2C). Importantly, *KRT7*, *CEACAM6* and *NAPSA* are expressed in only 11%–30% of EPCAM‐negative TCs implying routine pathological analysis of NSCLC samples with these markers may inadvertently overlook a large number of NSCLC cells undergoing the EMT process. Extracellular matrix (ECM) genes *COL1A1*, *COL1A2*, *COL3A1* and *SPARC* were significantly enriched in 52%–65% of EPCAM‐negative TCs (Figure 2A,C) while minimal expression of the ECM genes was observed in 3%‐28% of EPCAM‐positive TCs. GO analysis of genes enriched in EPCAM‐positive TCs revealed enrichment for gene signatures including growth and cellular homeostasis, whereas gene signatures enriched in EPCAM‐negative TCs included wounding and wound healing (Tables S6–8) (Figure 2B).

**FIGURE 2:**
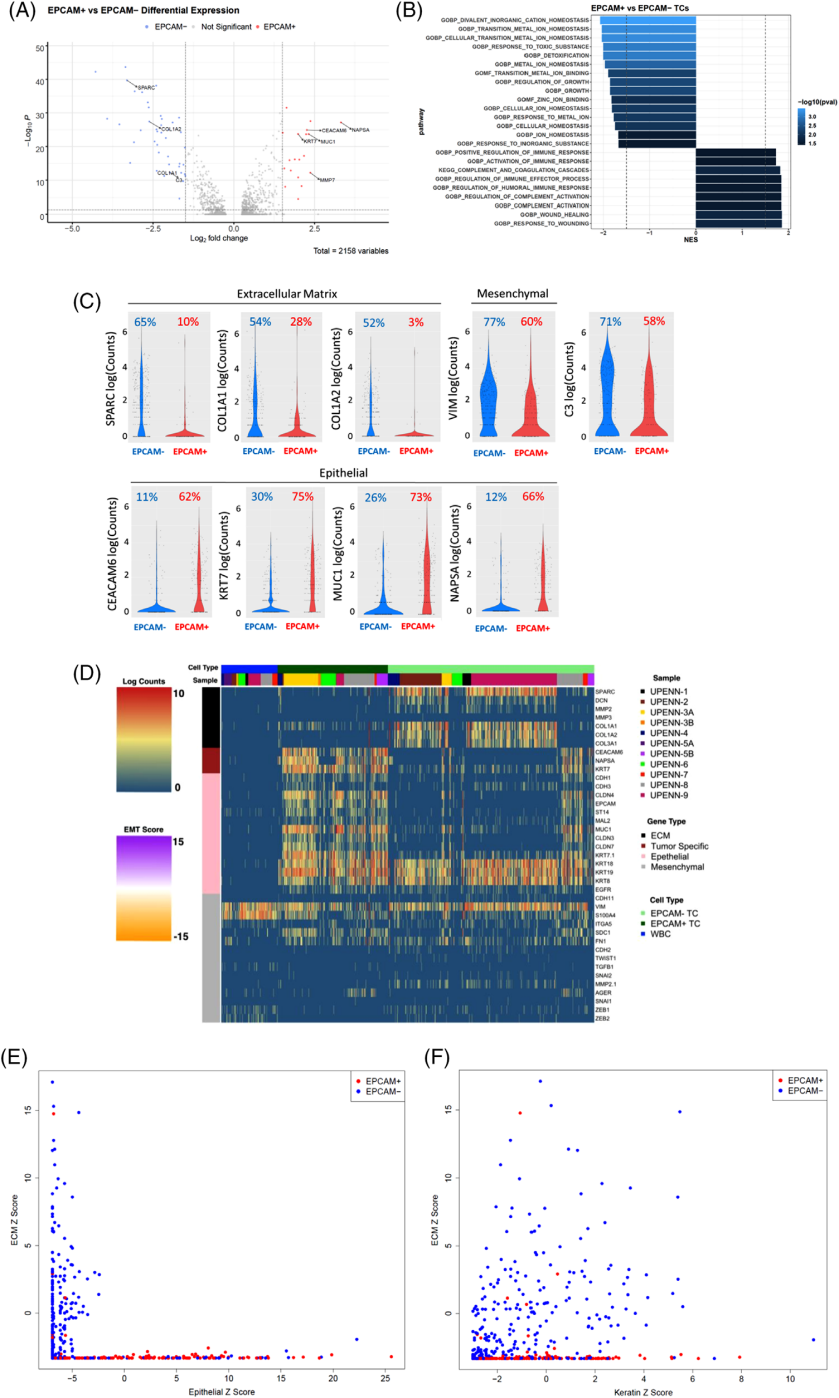
Characterization of EPCAM‐positive and EPCAM‐negative TCs and assessment of single‐cell heterogeneity of malignant pleural effusions (MPE) TCs. (A) Volcano plot of differentially expressed genes between EPCAM‐positive TCs and EPCAM‐negative TCs. Previously established non‐small‐cell lung cancer (NSCLC) tumour specific or epithelial to mesenchymal transition (EMT)/extracellular matrix (ECM) genes with log2‐fold change >1.5 and adjusted *p*‐value <0.05 are labeled (adjusted *p*‐value <0.05; log2‐fold change >1.5) in the volcano plot. (B) GO (gene ontology) pathways significantly enriched in EPCAM‐positive TCs compared to EPCAM‐negative TCs by gene set enrichment analysis (FDR < 0.05). (C) Violin plot of the log10 read counts of extracellular matrix‐associated genes *SPARC*, *COL1A1* and *COL1A2*, NSCLC specific genes *CEACAM6*, *KRT7*, *NAPSA*, cancer‐associated complement gene *C3*, mesenchymal gene *VIM* and epithelial gene *MUC1* in EPCAM‐positive and EPCAM‐negative TCs. Percentage of EPCAM‐negative and EPCAM‐positive cells expressing each gene are shown (D) Expression of EMT and ECM genes in MPE TCs and WBCs from NSCLC patients. Cell type and sample are shown on top of the heatmap. (E) Scatter plot of multi‐gene ECM Z score versus Epithelial Z score. (F) Scatter plot of multi‐gene ECM Z score versus Keratin Z score. An epithelial Z score was calculated by the sum of the log2 Z scores of 11 epithelial genes (*CEACAM6*, *NAPSA*, *CDH1*, *CDH3*, *CLDN4*, *CLDN3*, *CLDN7*, *EPCAM*, *ST14*, *MAL2* and *MUC1*), an ECM Z score was calculated by the sum of the log2 Z scores of seven ECM genes (*SPARC*, *DCN*, *MMP2*, *MMP3*, *COL1A1*, *COL1A2* and *COL3A1*) and a keratin Z score was calculated by the sum of the log2 Z scores of three keratin genes (*KRT18*, *KRT19* and *KRT8*). Scale bar of heatmap refers to log2 normalized UMI counts

We assessed the expression of a curated list of additional ECM, EMT and tumour specific genes to investigate single‐cell heterogeneity among TCs (Figure 2D). The majority of TCs expressed *KRT8*, *KRT18, KRT19*, and mesenchymal gene *VIM* with considerable heterogeneity in the expression of other epithelial and ECM genes. Next, we constructed a Z score to assess the relationship between the expression of epithelial, keratin and ECM genes. Epithelial (sum of the log2 Z scores of 11 epithelial genes), ECM (sum of the log2 Z scores of seven ECM genes) and keratin (sum of the log2 Z scores of three keratin genes) Z scores were calculated (genes listed in the figure legend). Scatter plot analysis verified that the expression of ECM and epithelial genes are largely mutually exclusive (Figure 2E). In contrast, EPCAM‐negative TCs with a high ECM Z score have a wide range of keratin expressions (Figure 2F).

Single‐cell heterogeneity within each patient sample was assessed by intracluster correlation coefficients (ICC score) using a curated gene set (Table S9). Lower ICC scores reflect higher heterogeneity. Eight of nine samples had high heterogeneity (ICC score range 0.012–0.261) and one sample (UPENN‐7) had low heterogeneity (ICC score 0.663) (Table S10). Thus, considerable single‐cell heterogeneity exists within patients.

Previously, we demonstrated that an EMT score calculated from RNA sequencing of bulk NSCLC tissue was significantly lower (more epithelial) in patients who respond to immunotherapy versus non‐responders.^9^ We sought to demonstrate the feasibility of measuring an EMT score from MPEs. The median single‐cell EMT score ranged from 4.61 for UPENN‐1 to ‐1.43 for UPENN‐5A, with considerable intra‐patient heterogeneity between the minimum and maximum single‐cell EMT scores (Figure 3A). All patients with a high EMT score (UPENN‐1, 7, 4, 2, and 9) had a high proportion of EPCAM‐negative TCs (range 76%–100%). In contrast, all patients with a low EMT score had a low proportion of EPCAM‐negative TCs (range = 26%–46%; Figure 3B). A similar inverse relationship between EMT score and EPCAM protein expression was detected at the single‐cell level (Correlation ‐0.322, *p*‐value 3.96e‐13) (Figure 3C,D, and Figure S3) in MPE TCs. A paired *t*‐test analysis revealed a significant difference between the EMT scores of EPCAM+ and EPCAM‐ TCs (*p*‐value < 0.0001) (Figure 3D).

**FIGURE 3:**
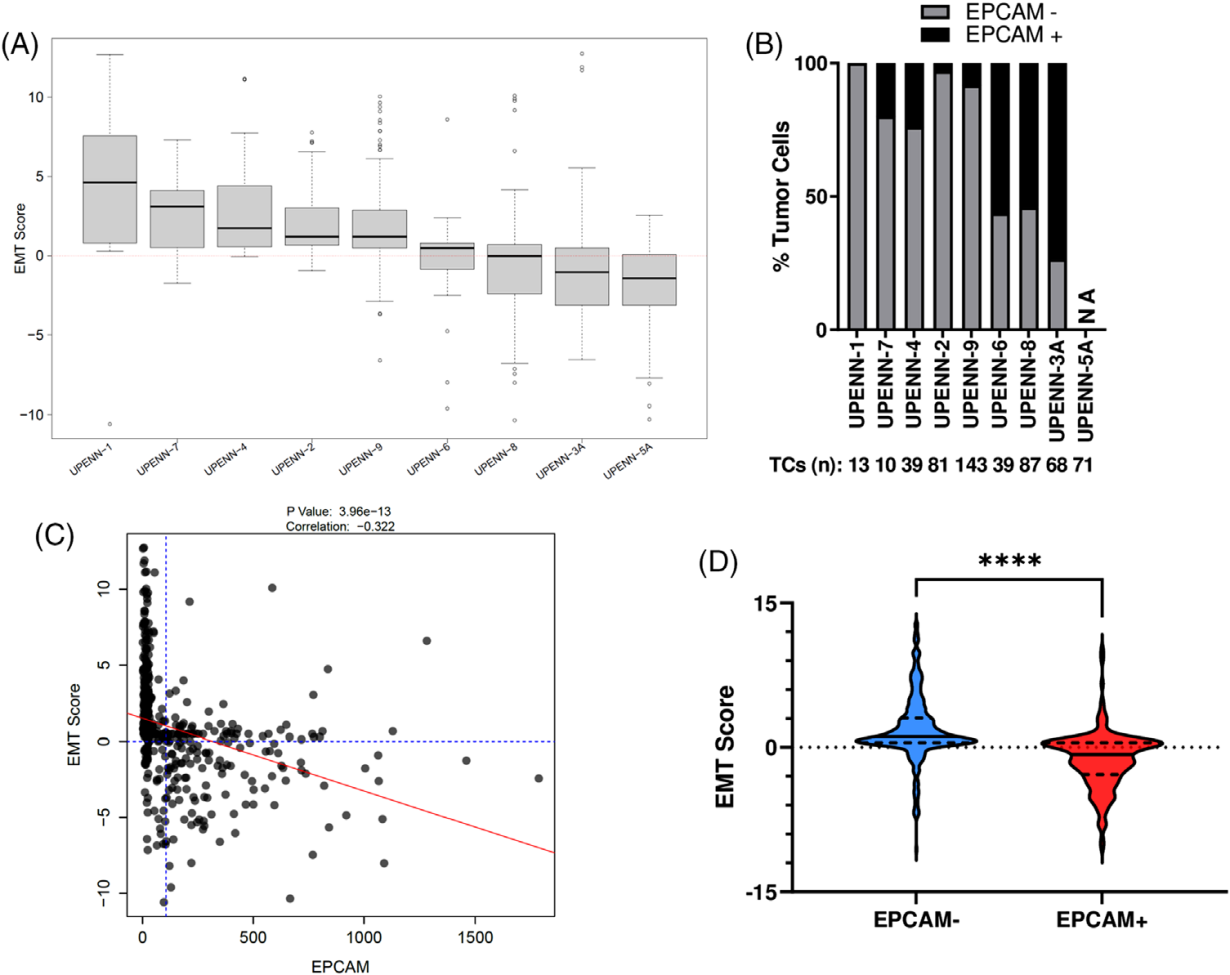
Single‐cell analysis of EMT in MPE TCs. (A) Box plots of EMT scores for single MPE TCs were calculated for each patient. The EMT score was calculated by the sum of the log2 Z scores of six established mesenchymal genes (*AGER*, *FN1*, *MMP2*, *SNAI2*, *VIM*, *ZEB2*) followed by subtracting the sum of the log2 Z scores of six established epithelial genes (*CDH1*, *CDH3*, *CLDN4*, *EPCAM*, *MAL2*, and *ST14*) B) Percentage of EPCAM‐positive and EPCAM‐negative TCs for each patient. The total number of TCs for each patient is shown below the patient number. (C) Linear regression was performed between EMT score and EPCAM protein expression for each MPE TC. A negative correlation was observed between the two variables. The relationship is statistically significant. D) Violin plot of the EMT score for EPCAM‐negative and EPCAM‐positive TCs. Dashed lines represent quartiles and solid line denotes the median score. Paired *t*‐test was utilized to assess significance (*p*‐value < 0.0001)

## CONCLUSION

Thus, through single‐cell transcriptional analysis, we show that the majority of MPE TCs did not express EPCAM and likely escaped detection in previous studies. The unbiased analysis of TCs allowed the identification of transcriptional differences in EPCAM‐positive and EPCAM‐negative TCs and uncovered significant intra‐patient heterogeneity in gene expression and EMT score. We establish the feasibility of an MPE liquid biopsy assay with a potential future diagnostic value as a liquid biopsy in NSCLC patients.

## CONFLICT OF INTEREST

Erica L. Carpenter received speaker's fees from Guardant Health, Imedex and Astra Zeneca, research funding from UHG, Janssen, Becton Dickinson and Merck, and an honorarium for membership in the advisory board from BMS. All funding outside the submitted work. Jeffrey C. Thompson has a consulting/advisory board role for AstraZeneca. Scott J. Bornheimer is an employee of and holds stock in BD Biosciences. Moen Sen, Ryan M. Hausler, Keely Dulmage, Taylor A. Black, William Murphy, Charles H. Pletcher, Ling Wang, Chang Chen, Stephanie S. Yee, Kara N. Maxwell, Ben Z. Stanger, and Jonni S. Moore have no competing interests.

## Supporting information


**Figure S1**. Box plots of the log10 read count for epithelial gene *EPCAM* and tumour‐specific genes *KRT8* and *KRT7*.

**Figure S2**. (A) Unsupervised hierarchical clustering of TCs and WBCs using genes that were significantly differentially expressed in TCs versus WBCs. EPCAM protein expression, cell type and sample are shown on top of the heatmap. (B) Volcano plot of differentially expressed genes between TCs and WBCs. Previously established NSCLC tumour specific or EMT genes with log2‐fold change >1.5 and adjusted *p*‐value <0.05 are labelled. (C) GO (Gene Ontology) pathways significantly enriched in PE TCs versus WBCs by gene set enrichment analysis. Normalized enrichment score (NES) corrects for differences in enrichment scores between gene‐sets due to differences in gene‐set sizes and allows comparison of the scores of the different tested gene‐sets. (FDR < 0.05) Scale bar of heatmap refers to log2 normalized UMI counts.

**Figure S3**. Linear regression was performed between EMT score and EPCAM protein expression for MPE TCs for individual NSCLC patients. Correlation and statistical significance are denoted over each plot.

Table S1

Table S2

Table S3

Table S4

Table S5

Table S6

Table S7

Table S8

Table S9

Table S10

Supplementary information
