# Supplementary figures and images for: Transcriptional profiling of single tumour cells from pleural effusions reveals heterogeneity of epithelial to mesenchymal transition and extra‐cellular matrix marker expression
Source: Clin Transl Med. 2022 Jul 10;12(7):e888. doi: 10.1002/ctm2.888 (PMC9271990; doi:10.1002/ctm2.888)

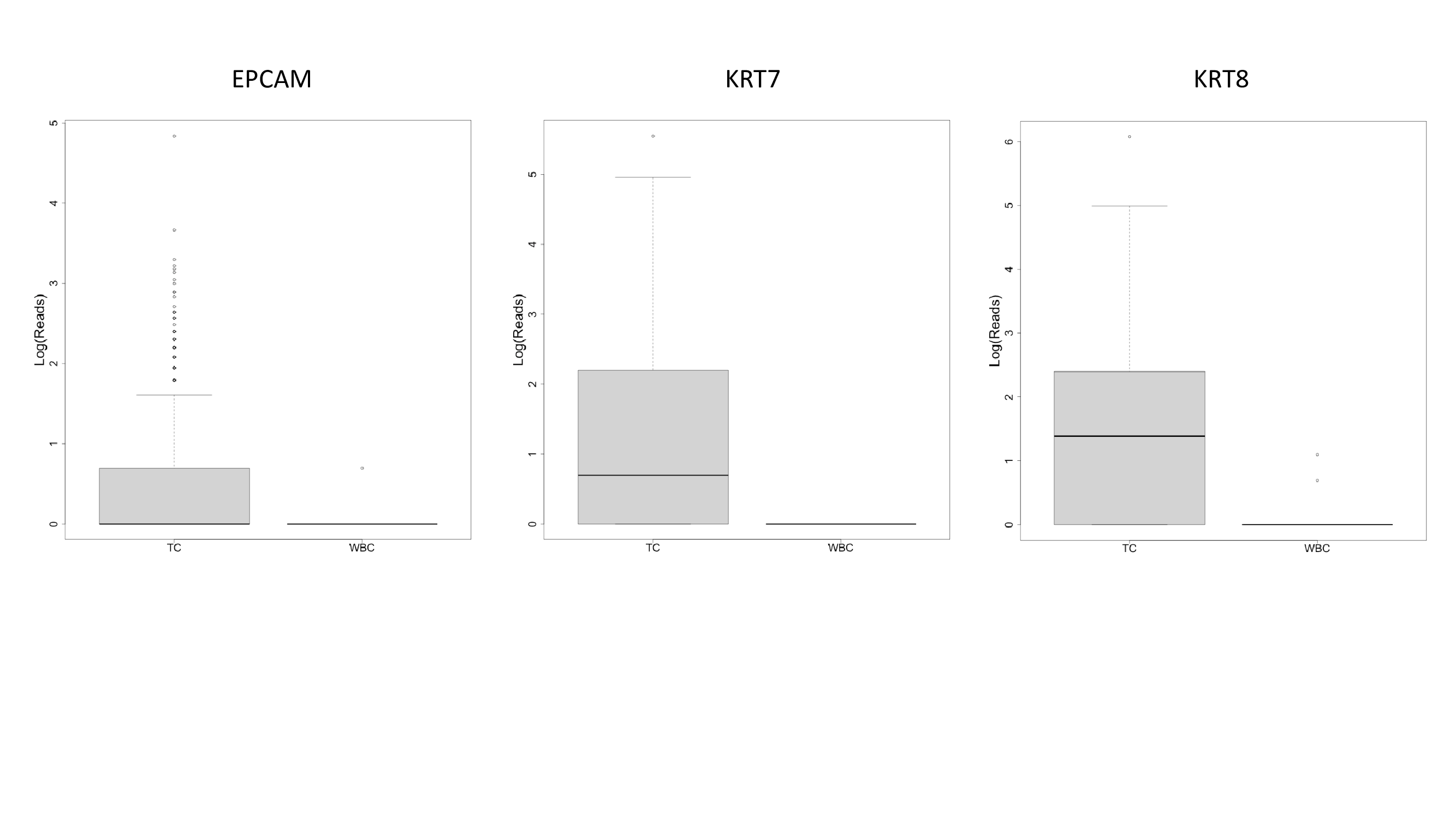

Supplement: Supplementary file 1 — Figure S1. Box plots of the log10 read count for epithelial gene EPCAM and tumour‐specific genes KRT8 and KRT7. [file CTM2-12-e888-s010.tiff]

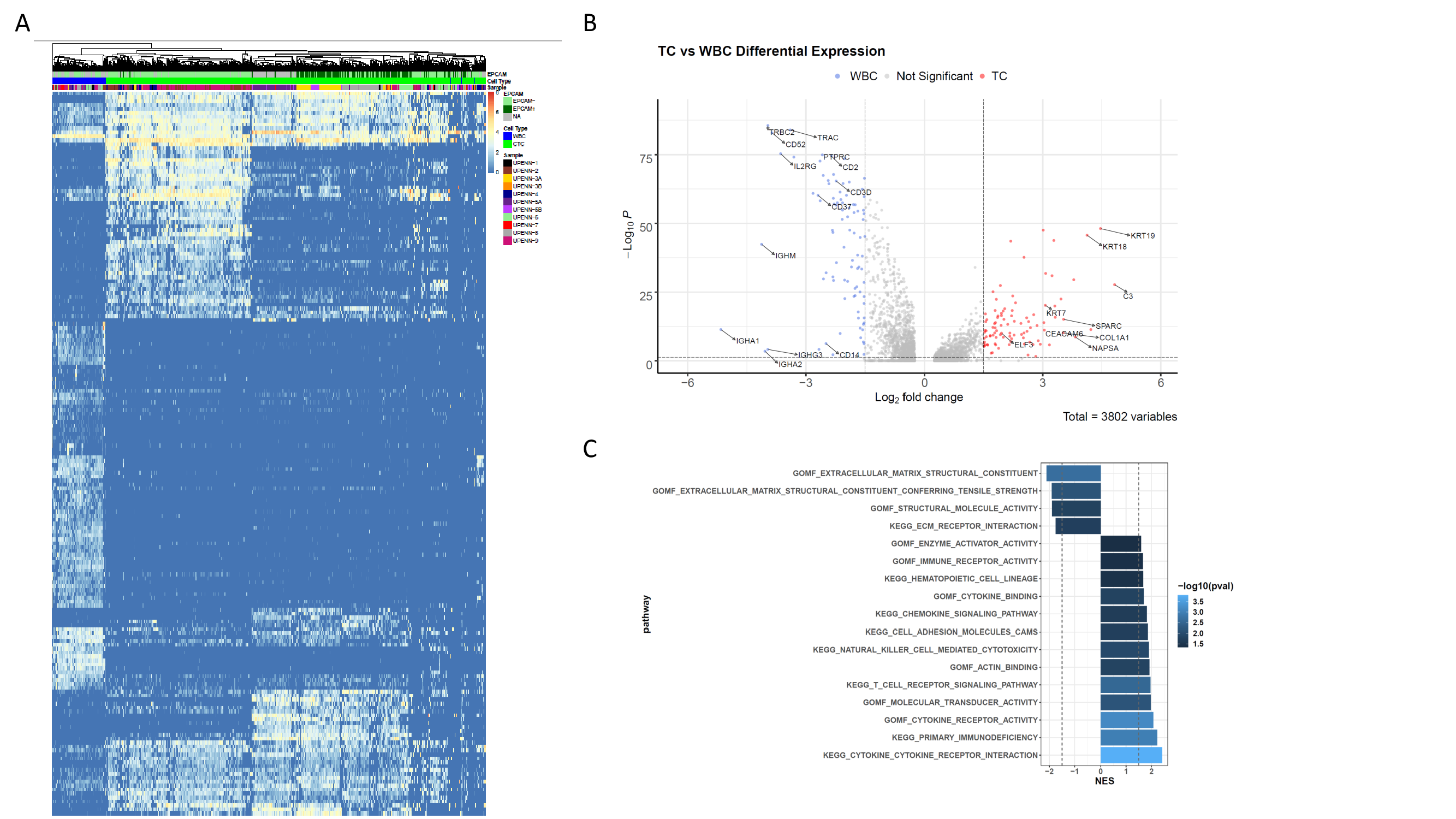

Supplement: Supplementary file 2 — Figure S2. (A) Unsupervised hierarchical clustering of TCs and WBCs using genes that were significantly differentially expressed in TCs versus WBCs. EPCAM protein expression, cell type and sample are shown on top of the heatmap. (B) Volcano plot of differentially expressed genes between TCs and WBCs. Previously established NSCLC tumour specific or EMT genes with log2‐fold change >1.5 and adjusted p‐value <0.05 are labelled. (C) GO (Gene Ontology) pathways significantly enriched in PE TCs versus WBCs by gene set enrichment analysis. Normalized enrichment score (NES) corrects for differences in enrichment scores between gene‐sets due to differences in gene‐set sizes and allows comparison of the scores of the different tested gene‐sets. (FDR < 0.05) Scale bar of heatmap refers to log2 normalized UMI counts. [file CTM2-12-e888-s006.tiff]

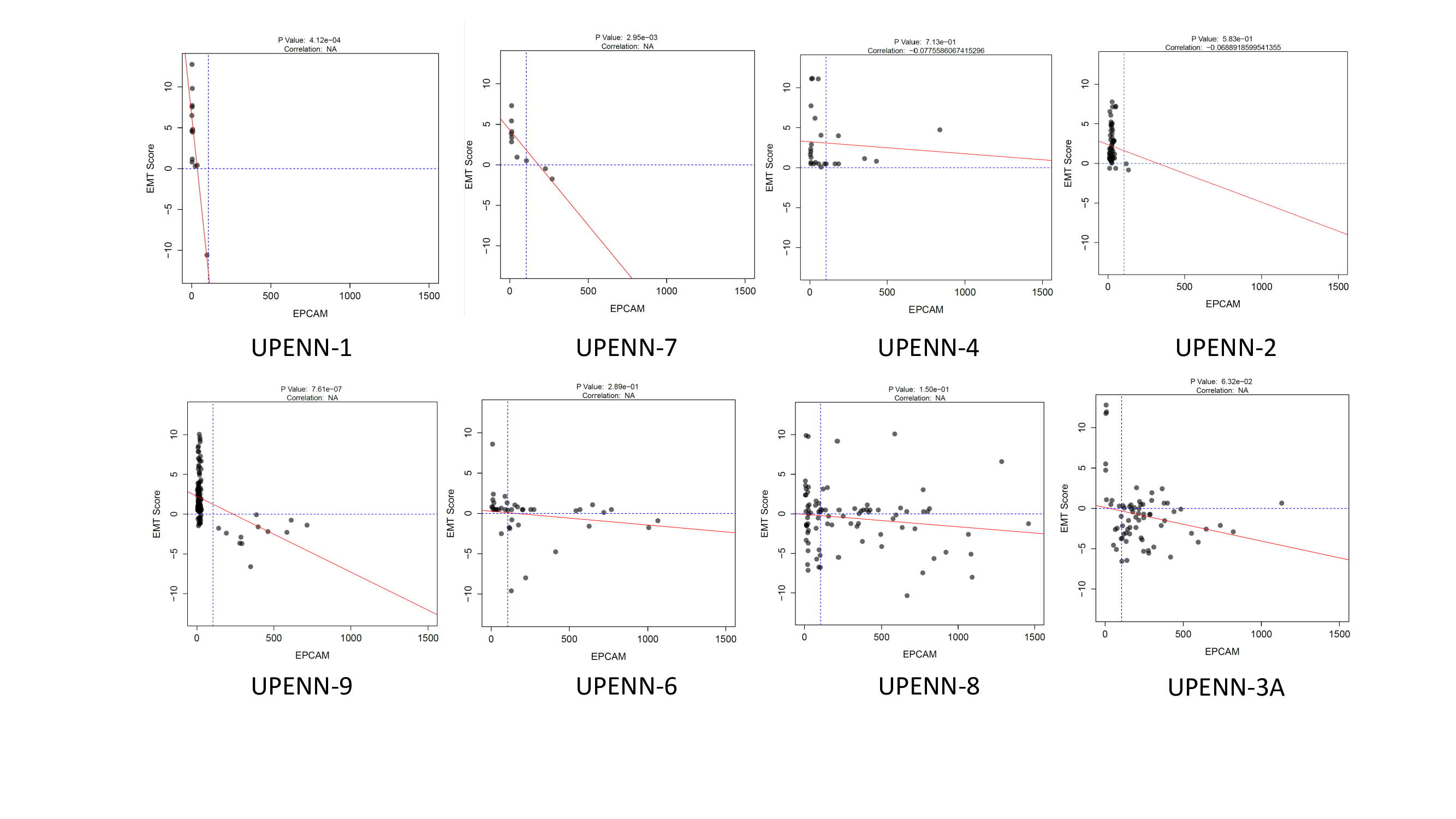

Supplement: Supplementary file 3 — Figure S3. Linear regression was performed between EMT score and EPCAM protein expression for MPE TCs for individual NSCLC patients. Correlation and statistical significance are denoted over each plot. [file CTM2-12-e888-s001.tiff]
