# Supplementary material for: Transcriptional profiling of single tumour cells from pleural effusions reveals heterogeneity of epithelial to mesenchymal transition and extra‐cellular matrix marker expression
Source: Clin Transl Med. 2022 Jul 10;12(7):e888. doi: 10.1002/ctm2.888 (PMC9271990; doi:10.1002/ctm2.888)
Supplement: Supplementary file 14 — Supplementary information [file CTM2-12-e888-s002.docx]

**2 MATERIALS AND METHODS**

**2.1 Patients**

This single-center, observational study was conducted at the Hospital of the University of Pennsylvania from July 2020 to April 2021 and was approved by the University’s Institutional Review Board. All nine subjects had a history of NSCLC and underwent sampling of a pleural effusion at our institution. A malignant pleural effusion was defined by the identification of malignant cells by cytologic analysis. Eleven malignant pleural effusion (MPE) specimens were collected from nine patients with metastatic lung adenocarcinoma at the Hospital of the University of Pennsylvania between July 2020 and April 2021 (Table 1). For patients UPENN-3 and UPENN-5, a second pleural effusion was sampled 127 and 170 days after the first sample respectively.

Baseline demographics and clinical variables, such as tissue driver mutation status, smoking status, treatment regimens and pathology results were obtained by chart review of the electronic medical records and clinical tissue next-generation sequencing. All patients had stage IV disease at the time of thoracentesis, and 67% of the patients were current or former smokers (Table 1). Eight of nine patients had an oncogenic driver mutation detected by tissue sequencing, including six *EGFR* mutations, one *BRAF* mutation and one *KRAS* mutation. Eight patients (89%) were white, one patient (11%) was Asian and all but one patient were female (89%). Two patients were receiving immunotherapy, five patients were receiving tyrosine kinase inhibitors (TKIs) and two patients were receiving chemotherapy at the time of their initial pleural effusion sampling.

**2.2 Malignant pleural effusion sample collection and preparation**

Pleural fluid (2-4 mL) was collected following thoracentesis. One ml of the sample at a time was stained with CD45-PE (Clone HI30) (BD Biosciences, CA, Cat: 555483) and EPCAM-FITC (Clone EBA-1) (BD Biosciences, CA, Cat: 347197) at 1:5 dilution at room temperature for 20 minutes with gentle shaking. To deplete WBCs, this was followed by incubation with BD IMAG^TM^ anti-PE magnetic particles (BD Biosciences, CA, Cat:557899) at 1:10 dilution for 20 minutes at room temperature with shaking. Then, one of three lysis conditions was applied for 10 minutes with gentle shaking based on the appearance of the MPE. Samples with no redness had no lysis buffer added, samples with a light red appearance had 1X volume lysis buffer added, and samples with a deep red appearance had 2X volume lysis buffer added (G-Biosciences, MO, Cat: 786-672).. A separate 1 ml aliquot of pleural effusion sample that was stained for CD45 and EPCAM but not incubated with anti PE-CD45 magnetic beads was used to sort CD45+ WBC pools. Prior to analysis on the flow cytometer, DAPI (4′,6-Diamidino-2-Phenylindone) was added at a final concentration of 1 μg/ml to allow for dead cell exclusion. MPE samples were processed through the pre-enrichment platform that employs magnetic bead-based white blood cell (WBC) depletion followed by acoustic focusing to wash away lysed RBCs resulting in enrichment of TCs. Enriched TCs were then analyzed and sorted on the BD Influx^TM^ cell sorter connected in-line with the pre-enrichment platform.

**2.3 Single cell index sorting**

MPE analysis and single cell sorting were performed on the BD Influx^TM^ cell sorter using BD FACS^TM^ Sortware software. We optimized a negative-selection gating strategy that did not rely on cell surface expression of EPCAM (Figure 1A). A gate was established for CD45-negative, DAPI-negative live cells larger than WBCs, as determined by forward scatter (FSC), and utilized to index sort single cells into a chilled 96 well BD™ Precise WTA single cell encoding plate containing lysis buffer, molecule-specific barcodes (Molecular Index), and sample barcodes (BD Biosciences, CA, Cat: 634100). 10-15 WBC cell pools were also index sorted into at least 4 wells per plate to serve as biological controls from an aliquoted tube of sample that was not incubated with anti PE-CD45 magnetic beads. Index sorting records the flow cytometric phenotype of each sorted cell and allows for retrospective correlation of phenotypic and molecular profiles at the single cell level. 3-7 wells per plate were left blank to serve as technical controls for downstream transcriptional analysis. After index sorting, plates were sealed with a foil cover, vortexed and centrifuged briefly and frozen at -80°C until further analysis.

**2.4 Whole transcriptome RNA sequencing**

Libraries were prepared for sequencing using the BD^TM^ Precise WTA Single Cell kit according to manufacturer’s instructions. Briefly, cDNA was synthesized in the wells of the 96-well BD Precise plates containing sample barcodes before being pooled together to generate Illumina compatible sequencing libraries with the Precise WTA Library Index Primers. To multiplex the libraries for sequencing, Illumina indexed adapters were incorporated at the end of each library prep to enable sequencing of 4-6 WTA Precise libraries using a NextSeq500 Mid Output v2 300 cycle sequencing kit (Illumina, CA, Cat. FC-404-2003) or 10-12 WTA libraries using a High Output v2 300 cycle sequencing kit (Illumina, Cat. FC-404-2004). The concentrations of amplified libraries were measured using Qubit dsDNA HS Assay Kit (Thermo Fisher Scientific, MA, Cat: Q32854), and the size and purity of the library was checked using a High Sensitivity D5000 ScreenTape (Agilent Technologies, CA, Cat. 5067-5593, 5067-5592) on the Agilent TapeStation. All the libraries were diluted to 2 nM and pooled together with 20% Phix before further diluting to 1.25 PM and loading onto the Illumina NextSeq500 System for sequencing with 2 x 75 bp paired-end reads.

FASTQ files from Illumina sequencing were processed with the BD™ Precise Whole Transcriptome Assay Analysis Pipeline v2.0 on the Seven Bridges Genomics platform to demultiplex and map reads from the sequencing files and calculate unique Molecular Indexes (MI) for each target with built-in correction algorithms. UCSC hg19 was used as the reference genome, and Gencode 19 was used for transcriptome annotation.

The RNA quality of cells was assessed for expression of GAPDH, PSMB4, PSMB2, REEP5 for TCs and PTPRC for WBCs. Cells with a molecular index read >1 in at least one of these housekeeping genes were included for downstream molecular analysis. In addition, cells were also filtered for a minimum number of counts, at least 2 standard deviations higher than the mean negative control value of blank wells.

**2.5 Data analysis**

Flow cytometric data was analyzed using FCS Express software. Data analysis including assessment of cluster distribution, principal component analysis (PCA), volcano plots and t-distributed stochastic neighbor embedding (t-SNE) analysis was performed using R statistical software and the Rtsne package. Where logged RNA counts are shown, these values include a pseudocount of +1. Hierarchical clustering was also used to categorize genes with distinct behaviors between different cell types. Significantly differentially expressed genes (DEGs) between samples were detected using the MAST package in R. Genes with a Bonferroni corrected p value of less than 0.05 were considered statistically significant. Gene set enrichment analysis was performed using the FGSEA R package^26^, utilizing Gene Ontology (GO) pathways.

Gene expression heatmaps were produced using the heatmap3 package in R (https://www.rdocumentation.org/packages/heatmap3/versions/1.1.9). All gene expression values underwent log base two transformation.

The epithelial to mesenchymal (EMT) score was previously established in NSCLC tumor tissue and generated by adding the sum of the log2 Z scores of 6 mesenchymal genes (AGER, FN1, MMP2, SNAI2, VIM, ZEB2) and subtracting the sum of the log2 Z scores of 6 epithelial genes (CDH1, CDH3, CLDN4, EPCAM, MAL2, and ST14). Positive EMT scores indicate more mesenchymal cells.

Z scores were created to summarize expression of groups of genes i.e., epithelial genes (CEACAM6, NAPSA, CDH1, CDH3, CLDN4, CLDN3, CLDN7, EPCAM, ST14, MAL2 and MUC1), ECM genes (SPARC, DCN, MMP2, MMP3, COL1A1, COL1A2 and COL3A1), and keratin genes (KRT18, KRT19 and KRT8). The Z scores for these groups were created by the sum of the Z scores for all the included genes. Z scores for each individual gene were determined by created using log base two transformed gene counts.

Intra cluster heterogeneity (ICC) values were determined per cluster via the following calculation: ICC = intercluster variance / (intracluster variance + intercluster variance). Intercluster variance is defined as the variance between the mean value in each cluster. Intracluster variance is defined as the variance of all values within a cluster. The genes used for ICC score calculation are denoted in table S9.
